# Genetic and epigenetic loss of miR-31 activates NIK-dependent NF-κB pathway in Adult T-cell Leukemia

**DOI:** 10.1186/1742-4690-8-S1-A128

**Published:** 2011-06-06

**Authors:** Makoto Yamagishi, Kazumi Nakano, Tadanori Yamochi, Ariko Miyake, Yayoi Kagami, Akihisa Tsutsumi, Aiko Otsubo, Seishi Ogawa, Atae Utsunomiya, Kazunari Yamaguchi, Kaoru Uchimaru, Toshiki Watanabe

**Affiliations:** 1 Graduate School of Frontier Sciences, The University of Tokyo, Tokyo, Japan; 2 Faculty of Medicine, The University of Tokyo, Tokyo, Japan; 3 Department of Haematology, Imamura Bun-in Hospital, Kagoshima, Japan; 4 Department of Safety Research on Blood and Biologics, National Institute of Infectious Disease, Japan; 5 Institute of Medical Science, The University of Tokyo, Tokyo, Japan

## 

Although crucial roles of microRNA have begun to emerge, detailed studies with ATL patients have not been achieved. Using 40 primary ATL samples and 22 samples of normal CD4+ T-cells, we determined the microRNA signatures of ATL and revealed loss of miR-31, which has recently been reported as a metastasis-associated miRNA. All ATL cases invariably showed undetectable or very low levels of miR-31, clearly implying that miR-31 loss is involved in ATL development.

As a novel miR-31 target gene, we identified NF-κB inducing kinase (NIK) that plays central roles in noncanonical signaling and constitutive activation of NF-κB in various cancers, including ATL. Restoration of miR-31 downregulated the levels of NIK and NF-κB activity, resulting in reduction of malignant phenotypes, containing proliferative index, anti-apoptosis, and chemotaxis in ATL cells. Furthermore, lentivirus-introduced miR-31 could induce strong apoptosis in primary tumor cells freshly isolated from ATL patients, indicating pivotal functions of miR-31 as a tumor suppressor.

Global copy number profiling demonstrated that 21 cases out of 168 (12.5%) have genomic loss of 9p21 containing miR-31 region. Furthermore, expression profiling and ChIP assay showed requirement of overexpression of histone methyltransferase in epigenetic suppression of miR-31 and aberrant NF-κB activation in primary ATL cells. Knockdown of methyltransferase complex restored the miR-31 expression and consequently inhibited NIK-dependent NF-κB cascade. These findings illustrated that genetic and epigenetic abnormalities link to NF-κB activation through the loss of miR-31. Considering aberrant epigenomics associated with cancers, the emerging relationship provides us a conceptual advance in understanding the broad-acting oncogenic signaling.

